# Identification of Cultured and Natural *Astragalus* Root Based on Monosaccharide Mapping

**DOI:** 10.3390/molecules200916466

**Published:** 2015-09-11

**Authors:** Ke Li, Xia Hao, Fanrong Gao, Guizhen Wang, Zhengzheng Zhang, Guanhua Du, Xuemei Qin

**Affiliations:** 1 Modern Research Center for Traditional Chinese Medicine, Shanxi University, No. 92, Wucheng Road, Taiyuan 030006, Shanxi, China; E-Mails: like@sxu.edu.cn (K.L.); wx2010haoxia@126.com (X.H.); gaofanrong@163.com (F.G.); wangguizhen@163.com (G.W.); zhangzzsxu@163.com (Z.Z.); dugh@imm.ac.cn (G.D.); 2 College of Chemistry and Chemical Engineering, Shanxi University, No. 92, Wucheng Road, Taiyuan 030006, Shanxi, China; 3 Institute of Materia Medica, Chinese Academy of Medical Sciences, Beijing 100050, China

**Keywords:** *Astragalus* root, monosaccharide mapping, quality evaluation, cultured RA, natural RA

## Abstract

As the main substances responsible for immunomodulatory activity, saccharides can be used as quality indicators for *Astragalus* root (RA). Saccharide content is commonly determined by ultraviolet spectroscopy, which lacks species specificity and has not been applied in the Chinese Pharmacopoeia. Monosaccharide mapping based on trifluoroacetic acid (TFA) hydrolysis can be used for quantitative analysis of saccharide compositions. In addition, species specificity can be evaluated by analysis of the mapping characteristics. In this study, monosaccharide mapping of soluble saccharides in the cytoplasm and polysaccharides in the cell wall of 24 batches of RA samples with different growth patterns were obtained based on TFA hydrolysis followed by gas chromatography-mass spectrometry. Results indicated that the mapping and the molar ratios of saccharide compositions of the cultured and natural RA samples were different for both cytoplasm and cell wall. For example, the molar ratio of mannose and arabinose was more than 3.5:1 in cytoplasm in cultured RA, whereas the ratio was less than 3.5:1 in natural RA. This research not only lays a foundation for screening indicators for RA, but also provided new ways of evaluating the quality of Chinese medicinal materials in which saccharides are the main bioactive substances.

## 1. Introduction

Astragalus Radix (RA) is a common Chinese medicinal material that has been used for more than 2000 years, with the first record appearing in “Shennong Bencao Jing” [1]. RA has beneficial effects on the spleen and in treating diseases of deficiency of qi and blood [2]. The Chinese Pharmacopoeia (2010 edition) specifies that a certified RA product must contain dried root of *Astragalus membranaceus* (Fisch.) Bunge var. *mongholicus* (Bunge) Hsiao (MG) or *A. membranaceus* (Fisch.) Bunge (MJ) [3].

At present, two kinds of RA are commercially available, namely, traditional RA with natural growth periods of more than five years, and fast-growing cultured RA with growth periods lasting one to three years. Traditional natural RA is mainly distributed in Shanxi, Inner Mongolia, Gansu, and Shaanxi; the production of MG (5–8 years old) from Shanxi and Inner Mongolia are considered genuine medicinal materials, which are characterized by “thick and long roots, fewer branches, mealiness, strong bean flavor, and sufficient sweetness” [4]. Cultured RA of MG grown for two to three years is mainly distributed in Gansu and Ningxia, and cultured RA of MJ grown for one year is mainly distributed in Hebei and Shandong; these RAs are characterized by “short roots, more branches, hard texture, and tastelessness”, compared with the traditional natural RA [5,6,7].

As the standard to evaluate the quality of the RA, the Chinese Pharmacopoeia (2010 edition) specifies that the content of Astragaloside ІV shall not be less than 0.04%. By determining the content of Astragaloside IV of 59 batches from different growth patterns, growth years, and commercial grades of RA samples, Tu *et al.* found that the content of Astragaloside IV decreases with the increase of growth years and commercial grades; in addition, cultured RA contains more Astragaloside IV than traditional natural RA [8,9,10].

The above finding indicates that under the current quality evaluation index of RA, the quality of cultured RA is similar to that of natural RA. However, the international market does not accept cultured RA, and some veteran TCM physicians still use natural RA. These conflicts show that the current quality evaluation method cannot be used for accurate assessment of the quality of RA. Therefore, a more rational evaluation method is urgently needed for the development of genuine medicinal materials.

In Traditional Chinese Medicine RA is believed to be a good medicine for “invigorating qi” and effective for treating deficiency of qi and blood [1,2,7]. An experimental and clinical study showed that RA can significantly increase immunity with a variety of active anti-inflammatory, anti-cancer, and anti-viral properties [11]. Numerous studies have shown that saccharides are the main substances responsible for immunomodulation, and different saccharides have different immunomodulatory activities [12,13]. Therefore, the content and variety of saccharides can be used as an index of quality evaluation for RA. At present, UV spectrophotometry is used to determine the total saccharide content for quality evaluation of RA. However, this method lacks specificity for RA, which limits its application in the Chinese Pharmacopoeia.

The successful establishment of peptide mapping technology using polypeptide hydrolase with different specificities has revolutionized the analysis of a biological system at protein level [14,15]. Similar to peptide mapping, saccharide mapping is developed based on saccharide hydrolysis [16,17,18,19]. Saccharide components in the cell are sequentially extracted according to the differences in the solubility of saccharide compounds in different solvents. Coupled with multivariate analysis, saccharide mapping of different medicinal materials from enzymatic hydrolysis or acid hydrolysis of sugar compounds extracts are utilized to reflect the specificity of herbs. The carbohydrate hydrolysis technology is the key factor of this technique. The specificity of the enzymatic hydrolysis method is prominent and can be used for identifying the varieties and linkages of saccharide [16,17]. However, more than 10 kinds of hydrolases are used in this method because of the complexity of sugar structure, thereby increasing its cost and complexity by employing different hydrolysis conditions. The acid hydrolysis method is mainly based on trifluoroacetic acid (TFA) hydrolysis with gas chromatography–mass spectrometry (GC-MS) [18]. This method not only can quantitatively analyze the variety and content of monosaccharides, but also has the advantages of simple operation, high specificity, and low cost. Guan *et al.* found differences in natural and cultivated *Cordyceps sinensis* by monosaccharide mapping based on acid hydrolysis [18]. Wu *et al.* distinguished the 12 kinds of different Ganoderma medicinal materials by the above method [19]. The technology laid foundations for the use of sugar compounds as a quality index for evaluation of TCMs.

In the present study, RA samples from 24 batches with different growth patterns were selected to elucidate the corresponding monosaccharide mapping, including soluble sugar in cytoplasm, polysaccharides in pectin, and hemicelluloses of cell wall, through sequential chemical extraction, TFA hydrolysis, acetylation, and GC-MS analysis. The components and classification indexes of the natural and cultured RA were compared and analyzed by quantitative monosaccharide mapping combined with multivariate and correlation analyses.

## 2. Results and Discussion

### 2.1. Establishment and Validation of Monosaccharide Mapping

**Figure 1:**
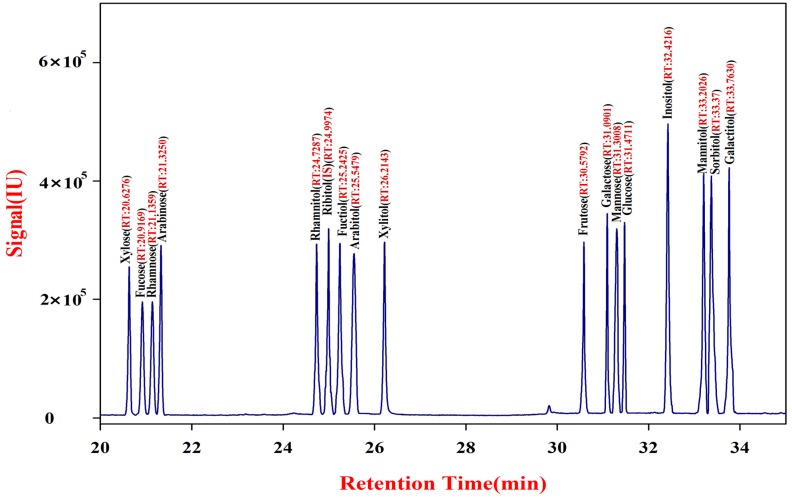
Typical chromatograms of 17 kinds of acetylated monosaccharide and alditol standards.

Figure 1 shows the total ion chromatogram of the acetylated products of 17 standard compounds of monosaccharides and alditols. The result shows that the standard equation of ribitol is lg*m_s_* = 7 × [lg(1.2 × *A_s_*) + lg2.4], with good linearity (R^2^ = 0.9921). Table 1 also shows that the blank recoveries of the acetylates of the sugar standard compounds are 91.11% to 99.04%, and the relative standard deviation is within 5%.

### 2.2. Analysis of the Monosaccharide Mapping in the Cytoplasm of RA

#### 2.2.1. Monosaccharide Mapping in the Cytoplasm of RA

The soluble sugars of RA include monosaccharides, alditols, disaccharides, oligosaccharides, polysaccharides, and glycoconjugates. To obtain the different monosaccharide mapping fractions, monosaccharides, alditols, disaccharides, and oligosaccharides in the cytoplasm were firstly extracted with 70% ethanol aqueous solution (Fraction A). Polysaccharides and glycoconjugates were subsequently extracted by water (Fraction B) because Fraction B can easily aggregate and precipitate in 70% ethanol aqueous solution [20]. Freeze-dried extracts were hydrolyzed by TFA and acetylated with acetic anhydride. The acetylated products were extracted by CH_2_Cl_2_, and the lower methylene chloride fractions were analyzed by GC-MS.

**Table 1:** Detection of 17 kinds of acetylated monosaccharide and alditol standards.

Name	Retention Time (min)	Recovery Rate (%)	RSD (%)	Detection Limit (mol∙L^−1^)	Correction Factor (f)
Xylose	20.6276	94.24	1.34	2.5 × 10^−8^	0.52
Fucose	20.9169	95.49	1.78	7.6 × 10^−9^	0.74
Rhamnose	21.1359	91.11	3.45	9.1 × 10^−8^	0.68
Arabinose	21.3250	97.78	0.97	1.1 × 10^−7^	0.84
Rhamnitol	24.7287	94.11	1.28	4.6 × 10^−8^	0.34
Ribitol	24.9974	99.04	3.22	3.4 × 10^−8^	1.00
Fucitol	25.2425	96.83	3.41	9.2 × 10^−8^	0.37
Arabitol	25.5479	94.77	2.25	5.8 × 10^−8^	0.71
Xylitol	26.2143	92.16	1.66	4.2 × 10^−8^	1.09
Fructose	30.5792	95.96	2.69	1.7 × 10^−7^	0.55
Galactose	31.0901	96.21	2.23	6.3 × 10^−8^	1.01
Mannose	31.3008	98.35	3.71	6.6 × 10^−8^	0.85
Glucose	31.4711	94.18	3.67	6.9 × 10^−8^	0.95
Inositol	32.4216	96.16	4.28	5.6 × 10^−9^	0.82
Mannitol	33.2026	96.60	1.43	5.5 × 10^−9^	0.84
Sorbitol	33.3700	95.51	2.19	1.2 × 10^−8^	0.70
Galactitol	33.7630	96.89	1.45	1.9 × 10^−8^	0.72

**Figure 2:**
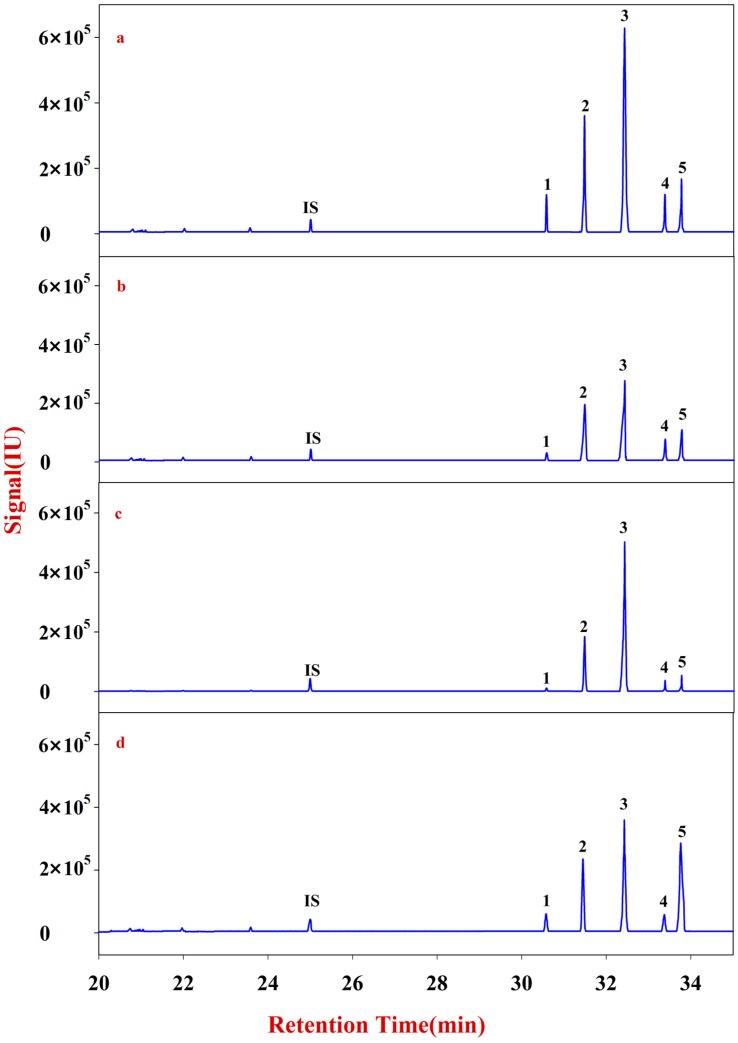
Monosaccharide mapping of Fraction A in RA samples in different growth patterns and species. **IS**: Ribitol (RT: 24.9974); **1**: Fructose (RT: 30.5792); **2**: Glucose (RT: 31.4711); **3**: Inositol (RT: 32.4316); **4**: Sorbitol (RT: 33.3729); **5**: Galactitol (RT: 33.7630). (**a**) monosaccharide mapping of Fraction A of natural MG; (**b**) monosaccharide mapping of Fraction A of cultured MG; (**c**) monosaccharide mapping of Fraction A of natural MJ; (**d**) monosaccharide mapping of Fraction A of cultured MJ.

Monosaccharide mapping of Fraction A of RA (Figure 2) shows that its composition mainly includes glucose, fructose, inositol, sorbitol, and galactitol (Table S1). The contents of glucose and fructose are significantly higher than those of the other three sugars. In addition, the total content of Fraction A in natural RA is significantly higher than that in cultured RA.

**Figure 3:**
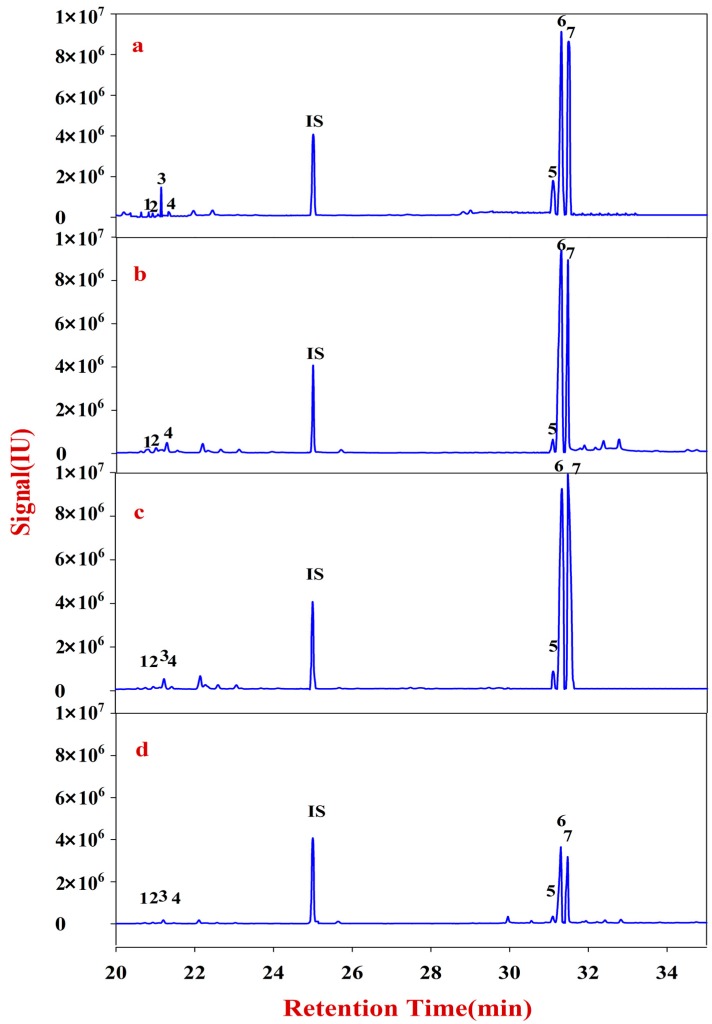
Monosaccharide mapping of Fraction B in RA samples in different growth patterns and species.**1**: Xylose (RT: 20.6276); **2**: Fucose (RT: 20.9169); **3**: Rhamnose (RT: 21.1359); **4**: Arabinose (RT: 21.3250); IS: Ribitol (RT: 24.9974); **5**: Galactose (RT: 31.0901); **6**: Mannose (RT: 31.3008); **7**: Glucose (RT: 31.4711). (**a**) monosaccharide mapping of Fraction B of natural MG; (**b**) monosaccharide mapping of Fraction B of cultured MG; (**c**) monosaccharide mapping of Fraction B of natural MJ; (**d**) monosaccharide mapping of Fraction B of cultured MJ.

Monosaccharide mapping of Fraction B of RA (Figure 3) shows that its composition mainly includes xylose, fucose, rhamnose, arabinose, galactose, mannose, and glucose (Table S2). These results are consistent with a previous report [21]. In addition, the contents of Fraction B in natural RA are also higher than those in cultured RA.

#### 2.2.2. Multivariate Statistical Analysis of the Monosaccharide Mapping of Fractions A and B of RA Cytoplasm

**Figure 4:**
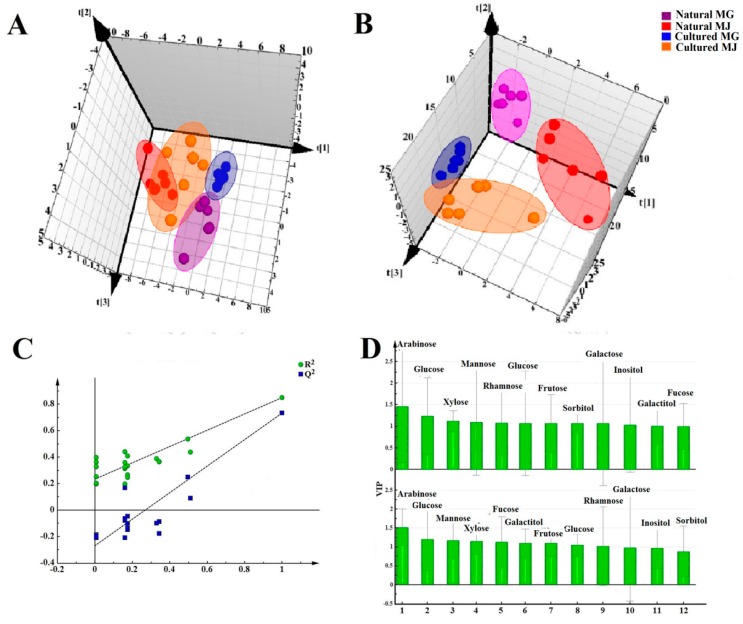
Multivariate statistical analysis of monosaccharides of Fractions A and B in the cytoplasm of RA samples in different growth patterns and species based on monosaccharide mapping. (**A**) PCA score plots; (**B**) PLS-DA score plots; (**C**) Permutations; (**D**) VIP plots.

Data of the contents of saccharides of Fractions A and B were subjected to various multivariate data analyses to determine the differences between natural and cultured RAs. We first employed PCA to highlight the differences or similarities between the natural and cultured RA samples. Figure 4A (PC1:0.621, PC2:0.218, PC3:0.092) shows a clear separation between natural and cultured RAs in the score plot, suggesting a substantial chemical difference between them. PLS-DA to classify the sample groups (Figure 4B). The model qualities were assessed with the total explained variables (R^2^X values) and the model predictability (Q^2^ values) followed by rigorous permutation tests (number: 200) [22].The high values of R^2^ (0.0710) and Q^2^ (−0.386) indicated good fitness of the model. All Q^2^ and R^2^ values were lower in the permutation test than in the real model, revealing high predictability and goodness of fit (Figure 4C). According to the VIP value (Figure 4D), the principal components to distinguish natural and cultured RAs are arabinose, glucose, mannose, xylose, and fucose of Fraction A, and galactitol, fructose, and glucose of Fraction B. Arabinose, glucose, xylose, mannose, and rhamnose of Fraction A, and glucose, fructose, sorbitol, galactose and inositol of Fraction B can be used to distinguish MG and MJ.

The contents of different monosaccharides of Fractions A and B in the cytoplasm of RA with different growth patterns and different species were subjected to *t*-test analysis, and the results are shown in Table 2 and Table 3.

**Table 2:** Comparison of *t*-test of the differential contents of monosaccharides of Fractions A and B in the cytoplasm of RA with different growth patterns.

		Varieties	MJ		MG	
	Content					
No.			Natural RA	Cultured RA	Natural RA	Cultured RA
1-Arabinose			2.923 ± 1.513	0.320 ± 0.118	9.191 ± 1.638	0.679 ± 0.356 ^▲▲^
1-Glucose			13.423 ± 3.748	0.451 ± 0.093 **	25.715 ± 4.684	3.219 ± 1.309 ^▲▲^
1-Mannose			13.019 ± 3.821	0.302 ± 0.081 **	20.366 ± 4.835	1.105 ± 0.222 ^▲▲^
1-Xylose			0.283 ± 0.119	0.174 ± 0.051	0.989 ± 0.315	0.708 ± 0.347
1-Fucose			0.215 ± 0.138	0.011 ± 0.001	0.053 ± 0.005	0.110 ± 0.062
2-Galactitol			0.193 ± 0.055	0.031 ± 0.012 *	0.337 ± 0.093	0.055 ± 0.022 ^▲^
2-Fructose			8.536 ± 0.508	6.787 ± 0.226 *	9.419 ± 0.299	7.986 ± 0.621
2-Glucose			8.536 ± 0.508	6.787 ± 0.226 *	9.419 ± 0.299	7.986 ± 0.621

“1-”represents Fraction A; “2-”represents Fraction B; * *p* < 0.05 different from natural RA of MJ; ** *p* < 0.01 significantly different from natural RA of MJ; ^▲^ *p* < 0.05 different from natural RA of MG; ^▲▲^ *p* < 0.01 significantly different from natural RA of MG; “MJ” represents the dried root of *A. membranaceus* (Fisch.) Bunge; “MG” represents the dried root of *A. membranaceus* (Fisch.) Bunge var. *mongholicus* (Bunge) Hsiao.

**Table 3:** Comparison of *t*-test of the differential contents of monosaccharides of Fractions A and B in the cytoplasm of different species of RA.

		Varieties	Natural RA		Cultured RA	
	Content					
No.			MJ	MG	MJ	MG
1-Arabinose			2.923 ± 1.513	9.191 ± 1.638 *	0.320 ± 0.118	0.679 ± 0.356
1-Glucose			13.423 ± 3.748	25.715 ± 4.684	0.451 ± 0.093	3.219 ± 1.309
1-Xylose			0.283 ± 0.119	0.989 ± 0.315	0.174 ± 0.051	0.708 ± 0.347
1-Mannose			13.019 ± 3.821	20.366 ± 4.835	0.302 ± 0.081	1.105 ± 0.222 ^▲▲^
1-Rhamnose			0.208 ± 0.099	0.506 ± 0.191	0.002 ± 0.000	ND
2-Glucose			8.536 ± 0.508	9.419 ± 0.299	6.787 ± 0.226	7.986 ± 0.621
2-Fructose			8.536 ± 0.508	9.419 ± 0.299	6.787 ± 0.226	7.986 ± 0.621
2-Sorbitol			0.112 ± 0.040	0.104 ± 0.038	0.007 ± 0.002	0.020 ± 0.006
1-Galactose			1.716 ± 0.659	3.176 ± 0.800	0.311 ± 0.134	1.681 ± 0.569 ^▲^
2-Inositol			0.519 ± 0.088	0.599 ± 0.109	0.036 ± 0.015	0.092 ± 0.357

“1-”represents Fraction A; “2-”represents Fraction B. * *p* < 0.05 different from natural RA of MJ; ^▲^ *p* < 0.05 different from cultured RA of MJ; ^▲▲^ *p* < 0.01 significantly different from cultured RA of MJ; “MJ” represents the dried root of *A. membranaceus* (Fisch.) Bunge; “MG” represents the dried root of *A. membranaceus* (Fisch.) Bunge var. *mongholicus* (Bunge) Hsiao. ND: Not detected.

Table 2 illustrates differences in content levels of Fraction B, including galactitol, fructose, and glucose, of RA in different growth patterns, as well as significant differences in the content levels of Fraction A, including arabinose, glucose, and mannose. The content of arabinose contributes larger than the glucose and mannose, distinguishing the differences between natural and cultured RA (as shown in Figure 4D). Therefore, the molar value of arabinose was set as 1; the molar ratio of glucose and mannose to arabinose are shown in Table 4. The ratio of the amount of mannose and glucose to arabinose ranges from 0.8 to 10.1. The ratio of the amount of mannose to arabinose was more than 3.5:1, and the ratio of the amount of glucose to arabinose was higher than 4:1 in cultured RA (for MG and MJ). The ratio of the amount of mannose to arabinose was less than 3.5: 1, and that of glucose and arabinose was below 4:1 in natural RA (for MG and MJ). Therefore, these ratios may be used to distinguish the growth patterns of RA.

**Table 4:** Molar ratio of different monosaccharides of Fractions A and B in the cytoplasm in natural and cultured RA.

Varieties	MG				MJ			
	No.	Arabinose	Mannose	Glucose	No.	Arabinose	Mannose	Glucose
Cultured RA	1	1	8.13	8.08	13	-	-	-
	2	1	3.72	4.36	14	1	7.03	7.13
	3	1	6.99	7.00	15	1	5.63	5.86
	4	1	8.04	10.16	16	1	5.26	5.18
	5	1	8.13	8.08	17	1	5.66	5.99
	6	1	4.92	8.47	18	-	-	-
Natural RA	7	1	2.74	3.18	19	1	2.18	2.20
	8	1	1.45	2.36	20	1	2.43	2.39
	9	1	3.15	4.54	21	1	1.83	2.06
	10	1	2.94	3.51	22	1	1.55	1.63
	11	1	0.81	1.10	23	1	2.93	3.07
	12	1	1.18	2.71	24	1	2.44	2.55

“-”arabinose is undetected and ratio does not exist in these samples; “MJ” represents the dried root of *A. membranaceus* (Fisch.) Bunge; “MG” represents the dried root of *A. membranaceus* (Fisch.) Bunge var. *mongholicus* (Bunge) Hsiao.

### 2.3. Analysis of the Monosaccharide Mapping of Pectin and Hemicellulose of RA Cell Wall

#### 2.3.1. Monosaccharide Mapping of Pectin and Hemicellulose of RA Cell Wall

Polysaccharides of pectin (Fraction C) and hemicellulose (Fraction D) can be extracted using endopolygalacturonase (EPG), Na_2_CO_3_, 1 M KOH, and 4 M KOH. To obtain different fractions for monosaccharide mapping, pectin in the cell wall were firstly extracted by EPG solution (Fraction C-1), and then extracted by Na_2_CO_3_ solution (Fraction C-2). Hemicellulose was subsequently extracted by 1 M KOH (Fraction D-1) and 4 M KOH solution (Fraction D-2). Freeze-dried extracts were hydrolyzed by TFA and acetylated by acetic anhydride. The acetylated products were extracted by CH_2_Cl_2_, and the lower methylene chloride fractions were analyzed by GC-MS.

**Figure 5:**
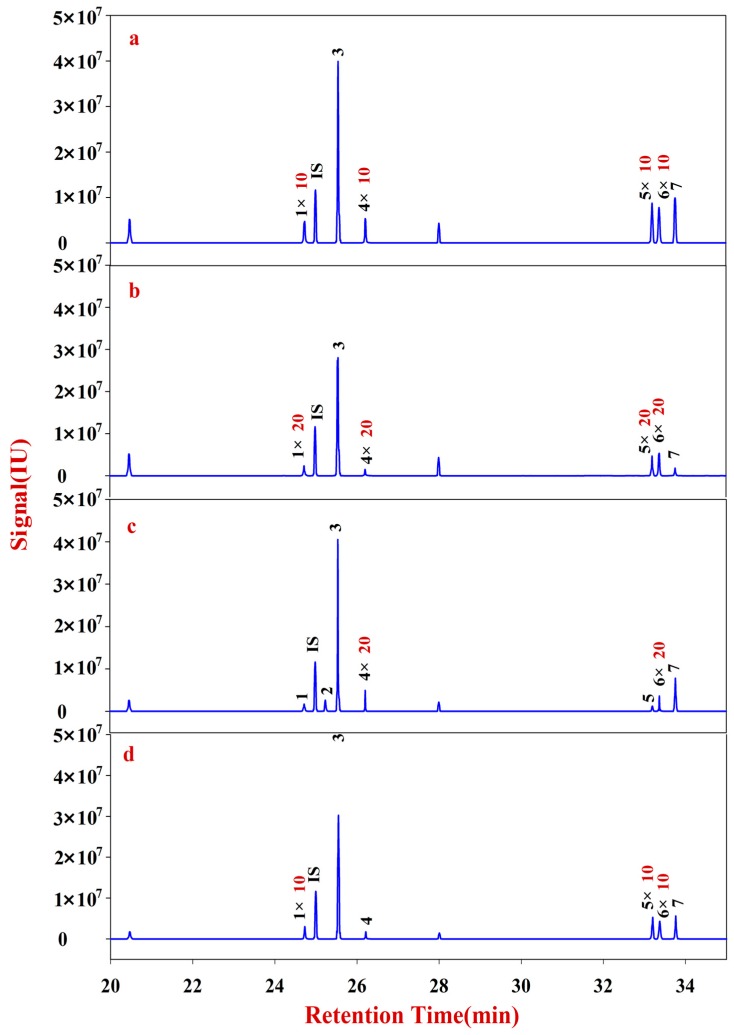
Monosaccharide mapping of Fraction C-1 of pectin in cell wall of RA samples in different growth patterns and species. **1**: Rhamnitol (RT: 24.7287); IS: Ribitol (RT: 24.9974); **2**: Fucitol (RT: 25.2425); **3**: Arabitol (RT: 25.5479); **4**: Xylitol (RT: 26.2143); **5**: Mannitol (RT: 33.2026); **6**: Sorbitol (RT: 33.3729); **7**: Galactitol (RT: 33.7630). (**a**) monosaccharide mapping of Fraction C-1 of natural MG; (**b**) monosaccharide mapping of Fraction C-1 of cultured MG; (**c**) monosaccharide mapping of Fraction C-1 of natural MJ; (**d**) monosaccharide mapping of Fraction C-1 of cultured MJ.

**Figure 6:**
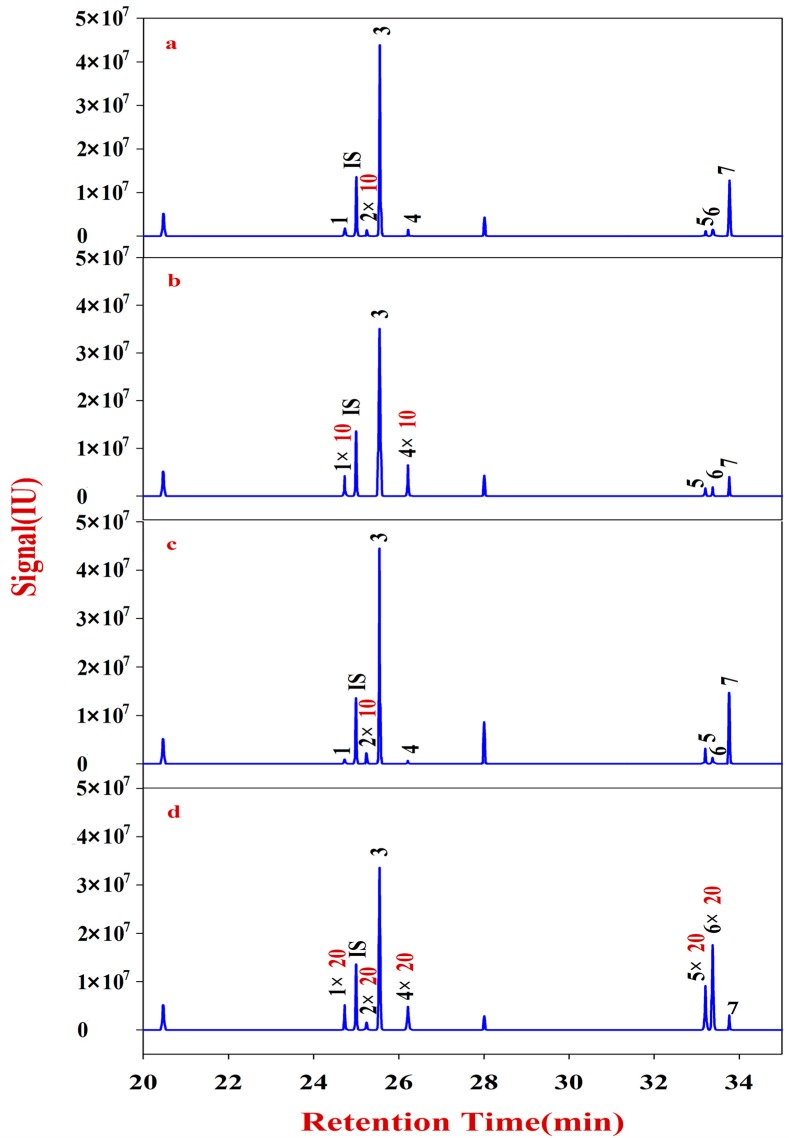
Monosaccharide mapping of Fraction C-2 of pectin in cell wall of RA samples in different growth patterns and species. **1**: Rhamnitol (RT: 24.7287); **IS**: Ribitol (RT: 24.9974); **2**: Fucitol (RT: 25.2425); **3**: Arabitol (RT: 25.5479); **4**: Xylitol (RT: 26.2143); **5**: Mannitol (RT: 33.2026); **6**: Sorbitol (RT: 33.3729); **7**: Galactitol (RT: 33.7630). (**a**) monosaccharide mapping of Fraction C-2 of natural MG; (**b**) monosaccharide mapping of Fraction C-2 of cultured MG; (**c**) monosaccharide mapping of Fraction C-2 of natural MJ; (**d**) monosaccharide mapping of Fraction C-2 of cultured MJ.

Monosaccharide mapping of Fraction C-1 (Figure 5) and Fraction C-2 (Figure 6) show that the compositions of Fraction C of RA mainly includes seven neutral monosaccharides, namely, xylose, fucose, rhamnose, arabinose, galactose, mannose, and glucose (Table S3). The content of arabinose is obviously higher than that of the others.

**Figure 7:**
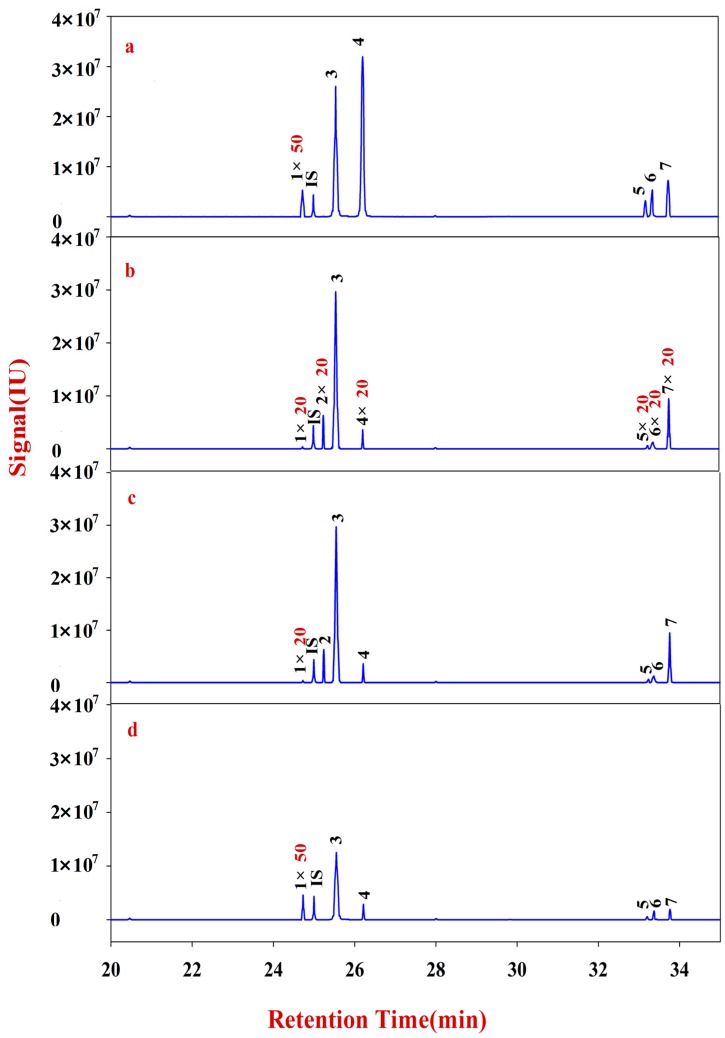
Monosaccharide mapping of Fraction D-1 of hemicellulose in cell wall of RA samples in different growth patterns and species. **1**: Rhamnitol (RT: 24.7287); **IS**: Ribitol (RT: 24.9974); **2**: Fucitol (RT: 25.2425); **3**: Arabitol (RT: 25.5479); **4**: Xylitol (RT: 26.2143); **5**: Mannitol (RT: 33.2026); **6**: Sorbitol (RT: 33.3729); **7**: Galactitol (RT: 33.7630). (**a**) monosaccharide mapping of Fraction D-1 of natural MG; (**b**) monosaccharide mapping of Fraction D-1 of cultured MG; (**c**) monosaccharide mapping of Fraction D-1 of natural MJ; (**d**) monosaccharide mapping of Fraction D-1 of cultured MJ.

**Figure 8:**
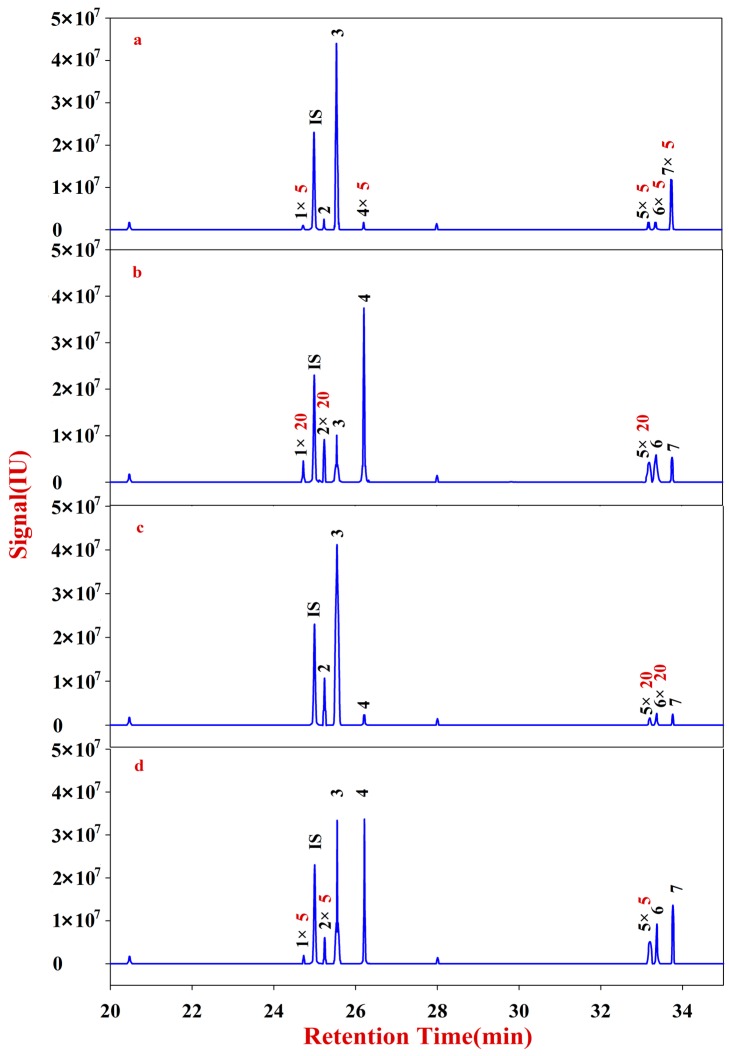
Monosaccharide mapping of Fraction D-2 of hemicellulose in cell wall of RA samples in different growth patterns and species. **1**: Rhamnitol (RT: 24.7287); **IS**: Ribitol (RT: 24.9974); **2**: Fucitol (RT: 25.2425) **3**: Arabitol (RT: 25.5479); **4**: Xylitol (RT: 26.2143); **5**: Mannitol (RT: 33.2026); **6**: Sorbitol (RT: 33.3729); **7**: Galactitol (RT: 33.7630). (**a**) monosaccharide mapping of Fraction D-2 of natural MG; (**b**) monosaccharide mapping of Fraction D-2 of cultured MG; (**c**) monosaccharide mapping of Fraction D-2 of natural MJ; (**d**) monosaccharide mapping of Fraction D-2 of cultured MJ.

Monosaccharide mapping of Fraction D-1 (Figure 7), Fraction D-2 (Figure 8) and the results (Table S4) show that the compositions of Fraction D in RA mainly have seven kinds of neutral monosaccharides: xylose, fucose, rhamnose, arabinose, galactose, mannose and glucose. The content of arabinose is obviously higher than that of others.

#### 2.3.2. Multivariate Statistical Analysis of Monosaccharide Mapping of Fractions C and D of RA

**Figure 9:**
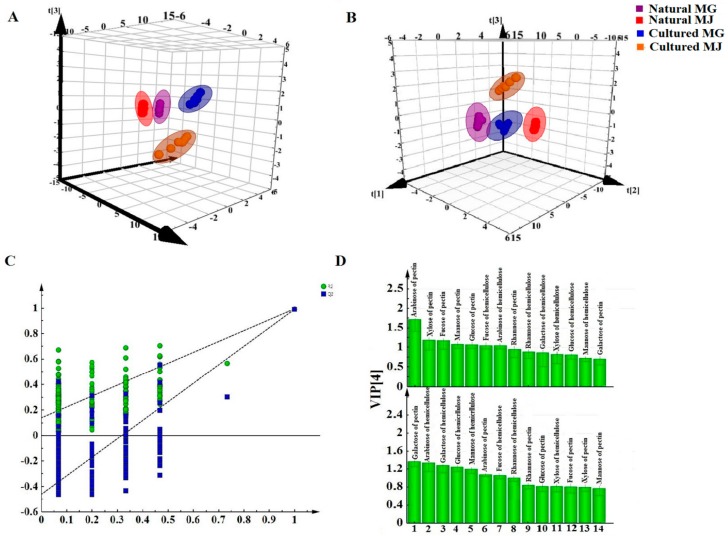
Multivariate statistical analysis of monosaccharides of Fractions C and D in cell wall of RA samples in different growth patterns and species based on Monosaccharide Mapping. (**A**) PCA score plots; (**B**) PLS-DA score plots; (**C**) Permutations; (**D**) VIP plots.

The PCA score plot (Figure 9A) (PC1:0.676, PC2:0.2, PC3:0.0975) for Fractions C and D parts showed clear distinction between the natural and cultured RA. PLS-DA to classify the sample groups (Figure 9B). The high values of R^2^ (0.0746) and Q^2^ (−0.477) indicated the good fitness of the model (Figure 9C). According to the value of VIP (Figure 9D), the principal components to distinguish natural RA and cultured RA are arabinose, xylose, fucose, mannose, and glucose of Fraction C, and fucose and arabinose of Fraction D. The principal components to distinguish MG and MJ are galactose and arabinose of Fraction C, and arabinose, galactose, mannose, and glucose of Fraction D.

The differential contents of monosaccharide of Fractions C and D in the cell wall of RA with different growth patterns and different species were subjected to t-test, and the results were showed in Table 5 and Table 6.

Table 5 illustrates significant difference in the levels of contents of Fractions C and D in cell wall, such as arabinose, xylose, fucose, mannose, and glucose of RA in different growth patterns. The content of arabinose contributes more than other monosaccharides to distinguish between natural and cultured RA (Figure 9D). Therefore, the molar value of arabinose of Fraction C was set as 1; the molar ratios of xylose, fucose, mannose, and glucose of Fraction C, and fucose and arabinose of Fraction D to the arabinose of Fraction C are shown in Table 7. The ratio range of the content of arabinose of Fraction D to that of Fraction C is 0.03–3.96. The ratio of the content of arabinose of Fraction D to that of Fraction C is more than 0.5:1 in cultured RA (MG and MJ). The ratio of the content of arabinose of Fraction D to that of Fraction C is less than 0.5:1 in natural RA (MG and MJ). These ratios may also be used to distinguish the growth pattern of RA.

**Table 5:** Comparison of *t*-test of the differential contents of monosaccharide of Fractions C and D in cell wall of different growth patterns of RA.

		Varieties	MJ		MG	
	Content					
No.			Natural RA	Cultured RA	Natural RA	Cultured RA
**3-Arabinose**			32.462 ± 0.969	4.562 ± 0.231 **	26.723 ± 1.168	1.333 ± 0.132 ^▲▲^
**3-Xylose**			0.203 ± 0.005	1.771 ± 0.059 **	0.136 ± 0.006	2.070 ± 0.198 ^▲▲^
**3-Fucose**			0.686 ± 0.016	0.030 ± 0.003 **	0.483 ± 0.030	0.007 ± 0.000 ^▲▲^
**3-Mannose**			0.019 ± 0.001	0.184 ± 0.003 **	0.042 ± 0.002	0.164 ± 0.031 ^▲▲^
**3-Glucose**			0.077 ± 0.000	0.790 ± 0.003 **	0.061 ± 0.001	0.416 ± 0.048 ^▲▲^
**4-Fucose**			0.012 ± 0.000	ND **	0.004 ± 0.000	ND ^▲▲^
**4-Arabinose**			12.442 ± 0.993	1.197 ± 0.044 **	4.034 ± 0.445	3.679 ± 0.129

“3-” represents Fraction C; “4-” represents Fraction D; ** *p* < 0.01 significantly different from natural RA of MJ; ^▲▲^ *p* < 0.01 significantly different from natural RA of MG; “MJ” represents the dried root of *A. membranaceus* (Fisch.) Bunge; “MG” represents the dried root of *A. membranaceus* (Fisch.) Bunge var. *mongholicus* (Bunge) Hsiao. ND: Not detected.

**Table 6:** Comparison of *t*-test of the differential contents of monosaccharides of Fractions C and D in cell wall of different species of RA.

		Varieties	Natural RA		Cultured RA	
	Content					
No.			MJ	MG	MJ	MG
**3-Galactose**			0.704 ± 0.009	0.502 ± 0.028 **	1.070 ± 0.032	0.464 ± 0.065 ^▲▲^
**4-Arabinose**			12.442 ± 0.993	1.197 ± 0.044 **	4.034 ± 0.445	3.679 ± 0.129
**4-Galacose**			1.273 ± 0.021	0.350 ± 0.003 **	0.374 ± 0.044	0.379 ± 0.007
**4-Glucose**			1.303 ± 0.020	0.355 ± 0.003 **	0.394 ± 0.045	0.420 ± 0.005
**4-Mannose**			0.107 ± 0.009	0.022 ± 0.001 **	0.0324 ± 0.004	0.0514 ± 0.002 ^▲▲^
**3-Arabinose**			32.462 ± 0.969	4.562 ± 0.231 **	26.723 ± 1.168	1.333 ± 0.132 ^▲▲^

“3-” represents Fraction C; “4-” represents Fraction D; ** *p* < 0.01 significantly different from MJ RA of natural RA; ^▲▲^ *p* < 0.01 significantly different from cultured RA; “MJ” represents the dried root of *A. membranaceus* (Fisch.) Bunge; “MG” represents the dried root of *A. membranaceus* (Fisch.) Bunge var. *mongholicus* (Bunge) Hsiao.

### 2.4. Cluster Analysis of the Monosaccharide Contents of RA Samples with Different Growth Patterns and Species Based on Monosaccharide Mapping

**Figure 10:**
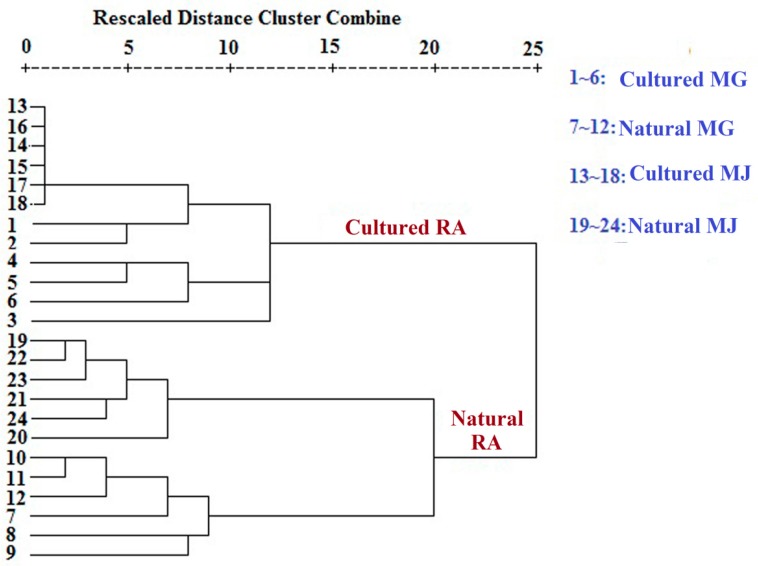
Dendrograms of hierarchical cluster analysis resulting from the contents of monosaccharide in RA samples in different growth patterns and species based on monosaccharide mapping.

The data of the contents of monosaccharide of Fractions A, B, C, and D in RA were combined, following data standardization by SPSS 16.0 cluster analysis (Figure 10). The figure shows that natural RA and cultured RA can be distinguished by multivariate statistical analysis of the monosaccharide contents of Fractions A, B, C, and D.

**Table 7:** Molar ratio of different monosaccharides of Fractions C and D in cell wall in natural and cultured RA samples.

Varieties	MG								MJ							
	No.	3-Arabinose	3-Xylose	3-Fucose	3-Mannose	3-Glucose	4-Fucose	4-Arabinose	No.	3-Arabinose	3-Xylose	3-Fucose	3-Mannose	3-Glucose	4-Fucose	4-Arabinose
Cultured RA	1	1	1.482	0.004	0.097	0.232	ND	2.441	13	1	0.371	0.007	0.031	0.128	ND	0.667
	2	1	1.643	0.005	0.136	0.309	ND	2.434	14	1	0.411	0.005	0.038	0.167	ND	0.957
	3	1	1.507	0.007	0.048	0.218	ND	3.960	15	1	0.388	0.006	0.034	0.144	ND	0.791
	4	1	1.554	0.004	0.115	0.266	ND	2.440	16	1	0.379	0.006	0.032	0.135	ND	0.725
	5	1	1.588	0.006	0.100	0.272	ND	3.059	17	1	0.399	0.006	0.036	0.155	ND	1.371
	6	1	0.434	0.006	0.036	0.153	ND	1.342	18	1	0.434	0.006	0.036	0.153	ND	1.342
Natural RA	7	1	0.005	0.017	0.001	0.002	0	0.036	19	1	0.006	0.019	0.000	0.002	0	0.428
	8	1	0.005	0.016	0.001	0.002	0	0.057	20	1	0.006	0.019	0.001	0.002	0	0.318
	9	1	0.005	0.017	0.001	0.002	0	0.045	21	1	0.006	0.020	0.001	0.002	0	0.393
	10	1	0.005	0.017	0.001	0.002	0	0.040	22	1	0.006	0.019	0.000	0.002	0	0.411
	11	1	0.005	0.016	0.001	0.002	0	0.050	23	1	0.006	0.019	0.001	0.002	0	0.356
	12	1	0.005	0.017	0.002	0.002	0	0.051	24	1	0.007	0.019	0.000	0.002	0	0.345

ND: Not detected. “3-” represents Fraction C; “4-” represents Fraction D.

### 2.5. Discussion

#### 2.5.1. Effect of the Content Difference of Fraction A of RA on Physiological State 

In this study, natural RA samples were selected from genuine producing areas, including the Hunyuan and Shanxi provinces of China. In these areas, the growing areas are mainly located on slopes with barren and porous soil, abundant sunshine during summer, arid (rainless) climate, large temperature differences between day and night, and cold winters. Natural RA grows for more than five years under such conditions. By contrast, cultured RA samples are cultivated on selected managed land and are harvested two years later. Natural RA suffers from drought, cold, nutrient deficiency, and other environmental stresses, whereas cultured RA is less affected by environmental stressors. 

Numerous studies have shown that plants usually resist environmental stress using the cells of the entire organism. Under stress, the morphology, physiological and biochemical properties, osmotic adjustment, plant hormone level, membrane, and the balance of active oxygen, stress proteins, and many other aspects of plants change in relation to plant water, photosynthesis, respiration, metabolism, and many other physiological processes [21,23,24,25,26]. Cytoplasmic free sugars and alditols components are the products of environmental stress during the growth of plants, and accumulation of these substances in the cells is related to osmotic adjustment, moisture retention, salinity resistance, low temperature and other adversity, and maintenance of the plant’s normal physiological function [23]. Therefore, natural RA contains more free sugars than cultured RA because of the environmental influence.

In addition, cultured RA has a better living environment and adequate nutrition during growth than natural RA. Thus, carbohydrates produced by photosynthesis are used for plant growth, such as for the construction of cytoskeleton, especially cellulose in the cell walls. However, with adverse circumstances and long growth periods, the absorbed energy material of natural RA is used to store energy by the synthesis of starch resist adverse environments. As a result, natural RA is characterized by “crude and long main roots, fewer branches, large mealiness, sufficient sweetness, and strong soybean odor.”

#### 2.5.2. Effect of the Content Difference of Fraction B of RA on Physiological State

Various polysaccharides and glycoconjugates, including glycoproteins, peptidoglycan, proteoglycans, and sugar esters, are present in the cytoplasms of plants. In addition, these macromolecules within plant cells serve as energy storage materials, which play important roles in plant seed germination, seedling growth, reproduction, stress response, and other life process [26,27,28,29]. For example, hydroxyproline-rich glycoprotein is involved in the process of resistance in plants, and arabinogalactan proteins play an important role in angiosperm fertilization. In the present study, the contents of polysaccharides and glycoconjugates in natural RA are higher than that in cultured RA, which is probably due to natural RA facing more complex living environment.

#### 2.5.3. Effect of the Content Difference of Fraction C and D of RA on Physiological State 

The plant cell wall is a highly ordered structure composed of different polysaccharides, proteins, and aromatic compounds. Polysaccharides constitute the main structural framework of the cell wall. Polysaccharides in plant cell walls include pectin, hemicellulose, and cellulose, among which cellulose serves as the main skeleton component [30]. As the first defense layer of the cell, the cell wall plays an important role in the plant response to external biotic and abiotic stressors, in which carbohydrates are the main factors to achieve its function. For example, a series of glycosyl hydrolases is activated during the interaction between the pathogenic bacteria and plant, proteoglycans on plant cell walls is degraded to small molecules glycoproteins, and trace glycoprotein can stimulate the plant to produce a strong antiviral response [31]. 

In summary, we speculate that natural RA is subjected to more environmental stresses than cultured RA, thereby inducing different levels of responses in cellular and molecular levels by their own defense mechanisms. These responses, including the formation of carbohydrate compounds with different structures for signal transduction, osmotic adjustment, and other physiological activities, are necessary to reduce the stress on the plants.

## 3. Materials and Methods

### 3.1. Plant Materials 

RA samples were obtained from two species of *A. membranaceus*. All plant materials were identified by Prof. Xue-Mei Qin, and the voucher specimens were deposited in the herbarium of the Modern Research Center for Traditional Chinese Medicine of Shanxi University. Detailed information on the samples is presented in Table 8. The 24 RA samples were dried, pulverized, and then stored at 20 °C in a vacuum desiccator until further analysis.

**Table 8:** List of RA plant materials.

Species	Cultivation Pattern	Regions	No.	Harvesting Time	Growth Year
MG	Cultured RA	Gs, Longxi County	1–2	2010.12	2
		Gs, Dangchang County	3	2010.12	2
		Sx, Daixian County	4	2011.1	2
		Sx, Yingxian County	5	2011.1	2
		Sx, Yanggao County	6	2011.1	2
	Natural RA	Ssx, Yulin	7–9	2011	5
		Sx, Hunyuan County	10	2011.11	≥5
			11–12	2011.1	5
MJ	Cultured RA	Hlj, Hulan County	13–15	2011.1	1
		Sd, Wendeng County	16–18	2011	1
	Natural RA	Hlj, Jiagedaqi	19–20	2011	5
		Hlj, Hulan County	21	2011.11	5
		Hlj	22–24	2011	5

### 3.2. Solvents and Chemicals 

Analytical-grade Na_2_CO_3_, KOH, acetic anhydride, NaBH_4_, and tris-(hydroxymethyl) methyl aminomethane were purchased from Fengchuan Chemical Co. Ltd. (Tianjin, China). Analytical-grade ethanol acetic anhydride, CH_2_Cl_2_, and acetone were purchased from Beijing Chemical Works (Beijing, China). TFA, dimethyl sulfoxide (DMSO), and 1-methylimidazole were obtained from Sangon Biotech Co., Ltd. (Shanghai, China). The 17 standard compounds of monosaccharides and alditols (d-glucose, d-galactose, d-mannose, d-fructose, d-xylose, d-arabinose, l-fucose, l-rhamnose, l-inositol, d-ribitol, sorbitol, galactitol, mannitol, xylitol, arabinitol, fucitol, and rhamnitol), were purchased from Sigma-Aldrich China (Shanghai, China). Pectinase (>500 µ/mg) from *Aspergillus* was purchased from Sangon (Shanghai, China). Type-I porcine a-amylase (250 µ/mg) was obtained from Sigma-Aldrich China.

### 3.3. Preparation of 17 Standard Monosaccharide and Alditol Acetylation Products and Methodology Validation

Derivatization of authentic standard compounds of soluble saccharides was done following the previously described method [32]. This method was concurrently used to generate GC-MS chromatograms of the 17 standard compounds of monosaccharides and alditols.

Authentic standard saccharide solution of ribitol (IS)-DMSO (1:10 *w*/*v*; 200 µL) was mixed with 150 μL of acetic anhydride and 30 μL of 1-methylimidazole into 10 mL glass centrifuge tubes and then left to stand for 10 min. Double-distilled H_2_O (ddH_2_O, 600 µL) was added to the tubes to remove excess acetic anhydride. Then, 200 μL of CH_2_Cl_2_ was added to the tubes. The tubes were centrifuged for 1 min to partition the organic phase. Finally, 1 μL of the lower methylene chloride fraction was injected for GC-MS analysis.

A series of standard solutions of ribitol in DMSO at seven levels including 0.5, 2.5, 6.25, 25, 50, 100 and 200 ng/μL was prepared for determining the standard equation. These solutions were derivatized as described in the manuscript and three replications of each level were performed. According to the following formula, the standard equation of internal standard ribitol and the relative correction factor for each standard compound was calculated using ribitol as an internal standard:

(1) $$\text{The}\;\text{standard}\;\text{equation}:\;\text{lg}m_{s}=a\text{lg}A_{s}+b$$

(2) $$\text{The}\;\text{relative}\;\text{correction}\;\text{factor}:f=(m_{s}\times {m_{i}}^{-1})\times (A_{i}\times {A_{s}}^{-1})$$

where *m_i_* and *m_s_* respectively stand for the detection masses of monosaccharide and inner standard of ribitol; *A_i_* and *A_s_* respectively stand for the peak areas of monosaccharide and inner standard of ribitol; f stands for the relative correction factor of monosaccharide to ribitol. We calculated the standard equation of ribitol, detection limit, and the recovery rate of each standard compound. 

### 3.4. GC-MS Detection Conditions

An ion trap MS system (Trace-GC Ultra) connected to a Trace-Plase Q Mass Selective Detector (Thermo Finnigan, San Jose, CA, USA) was used in the analysis of acetyl derivatives on a DB-5 MS fused silica capillary column (30 m × 0.25 mm, 0.25 μm film thickness, Agilent, Santa Clara, California, CA, USA) and with ionization of full scan and selected-ion monitoring (selected ion ± 1.0 mass unit). The oven temperatures were programmed as follows: initiation at 100 °C, gradual ramped to 180 °C (5 °C/min), and held for 1 min; 190 °C (1 °C/min) and held for 2 min; then increased to 220 °C (30 °C/min), holding for 2 min; then increased to 230 °C (1 °C/min), holding for 2 min; ramped to 280 °C (20 °C/min), and held for 10 min. The temperature at the injector port was 250 °C, and that at the detector port was 220 °C. Helium was used as the carrier gas at a constant flow rate of 1.0 mL/min. An Xcalibur 2.0 workstation was used for data acquisition and quantitative data processing.

### 3.5. Sequential Extractive Process of Fractions A and B of RA

The saccharide components in the cytoplasm of RA preparation were determined as previously described [18]. Briefly, 20 g of RA fine powder was placed into a 500 mL round-bottom flask, to which 200 mL of 70% ethanol aqueous solution was added for extraction. The solution was extracted at 100 °C for 1 h, cooled to room temperature, and then centrifuged. The supernatant was freeze-dried to obtain a powder of Fraction A. The residual powder was placed into a 500 mL round-bottom flask, to which ddH_2_O (1:80, *w*/*v*) was added. The solution was extracted at 100 °C for 3 h and then centrifuged. The extraction process was repeated, in which two times supernatant were combined, concentrated, and freeze-dried to obtain a powder Fraction B.

### 3.6. Alcohol Insoluble Residue (AIR) Preparation

AIR of RA was prepared as previously described [33]. The precipitate was placed into a 500 mL conical flask, to which 80% ethanol aqueous solution (1:100, *w*/*v*) was added. The reaction was placed in a water bath at 60 °C for 1 h, and then centrifuged at 3500 rpm for 10 min, the supernatant was discarded, and the above experiment was repeated several times until the supernatant was colorless. Acetone (1:10, *w*/*v*) was added to the conical flask. The solution was allowed to settle for some time, then the supernatant was discarded, and the residue dried in an oven at 45 °C. The residual powder was placed into a 500 mL conical flask, to which a buffer (1:100, *w*/*v*; 50 mM Tris-HCl, pH 7.0) containing type-I porcine α-amylase (20 units/mL) was added. The reaction was placed in a shaking table at 37 °C for 12 h, and then centrifuged at 3500 rpm for 10 min; the supernatant was discarded. The residue was washed for several times by ddH_2_O, and then centrifuged. The supernatant was discarded and acetone (1:10, *w*/*v*) was added to the residue. The solution was left to stand for some time and the supernatant was discarded. The residue was dried in an oven at 45 °C.

### 3.7. Sequential Extraction Process of Fractions C and D of RA

Portions of the Fraction C were solubilized from the cell walls of RA by each of the following sequential treatments: EPG and Na_2_CO_3_ as previously described [34]. Briefly, AIR fine power material (2 g) was weighed and placed into a 100 mL centrifuge tube, to which 70 mL of buffer (sodium acetate 50 mM, pH 5.0) containing pectinase (20 μ/mL) were added. The reaction was placed in a shaking table at 30 °C for 24 h, and then centrifuged at 3500 rpm for 10 min; the supernatant was removed and stored at 4 °C. The above steps were repeated for two or three times, and then the residue was washed with 20 mL ddH_2_O for three times. The supernatant and washes were combined in a 500 mL conical flask, which was placed in a water bath at 70 °C for 30 min. Then, the solution was run through a dialysis tubing (3500 molecular-weight cutoff), concentrated, and then lyophilized.

The residual walls were added with 70 mL of 50 mM Na_2_CO_3_ and 5 mM EGTA. The reaction was placed in a shaking table at 30 °C for 24 h, and then centrifuged at 3500 rpm for 10 min. The supernatant was removed and stored at 4 °C. The above steps were repeated two or three times, and then the residue was washed with 20 mL of ddH_2_O three times. The supernatant and washes were combined, neutralized with acetic acid, dialyzed, concentrated, and lyophilized.

After Na_2_CO_3_ extraction, the walls were treated sequentially with 1 M and 4 M KOH to solubilize Fraction D. The remaining residual walls after Na_2_CO_3_, extraction were incubated in 70 mL of 1 M KOH solution containing 1% NaBH_4_ at 30 °C for 24 h. Then, the wall suspension was centrifuged at 3500 rpm for 10 min, and the supernatant was removed and stored at 4 °C. The above steps were repeated two or three times, and then the residue was washed with 20 mL ddH_2_O three times. The supernatant and washes were combined, neutralized with acetic acid, dialyzed, concentrated and lyophilized.

The remaining residual walls after extraction with 1 M KOH were incubated in 70 mL of 4 M KOH solution containing 1% NaBH_4_ at 30 °C for 24 h. Then, the wall suspension was centrifuged at 3500 rpm for 10 min, and the supernatant was removed and stored at 4 °C. The above steps were repeated two or three times, and then the residue was washed with 20 mL of ddH_2_O three times. The supernatant and washes were combined, neutralized with acetic acid, dialyzed, concentrated, and lyophilized.

### 3.8. Hydrolization by TFA of Carbohydrates of RA 

Acid hydrolysis of RA was determined as described previously [34]. Fraction A powder (20 mg) was placed into a 10 mL centrifuge tube, to which 2 mL of 2 M TFA was added. The tube was sealed and placed in an oil bath at 110 °C for 1h. Then, after drying under a gentle steam of air, the sample was redissolved with 1 mL of methanol and then dried again. The experiment was repeated for three times, and 1 mL of internal standard solution ribitol (1:10, *w*/*v*) was added. Fractions B, C, and D powders (20 mg each) were separately placed into 10 mL centrifuge tubes. Hydrolysis products obtained by the above method were dissolved in 1 mL of ribitol (1:10, *w*/*v*).

### 3.9. Preparation of Carbohydrates of RA Acetylation Products

The solutions of Fractions A and B were transferred into 10 mL glass centrifuge tubes for derivatization. After drying, the samples were redissolved into 200 μL of CH_2_Cl_2_ for filtering. The reaction mixture (1 μL) was injected into the GC-MS for analysis. Fractions A and B of RA were quantitatively analyzed according to an internal standard.

The solutions of Fractions C and D were transferred into 10 mL glass centrifuge tubes, to which 500 μL of DMSO containing 2% NaHB_4_ was added. The reaction was placed in a shaking table at 40 °C for 90 min, neutralized with acetic acid, and then derivatized. After drying, the sample was redissolved into 200 μL of CH_2_Cl_2_ for filtering. The reaction mixture (1 μL) was injected into the GC-MS for analysis. Fractions C and D of RA were quantitatively analyzed according to an internal standard.

### 3.10. Statistical Analysis

Multivariate analyses for all data were performed using SIMCA-P 13.0. Correlation analyses for all data (mean ± SD) were performed using SPSS 16.0. Monosaccharides mapping obtained by SigmaPlot 12.0 were used to establish all figures.

## 4. Conclusions

In this study, monosaccharide mapping of Fractions A, B, C, and D of 24 batches of RA samples with different growth patterns were obtained based on TFA hydrolysis followed by GC/MS. Fractions A, B, C, and D were mainly composed of glucose, fructose, inositol, sorbitol, galactitol, xylose, fucose, rhamnose, arabinose, galactose, and mannose. Statistical analyses showed that the contents of monosaccharides in natural RA were higher than those of cultured RA. 

Monosaccharide mapping of RA by multivariate analysis showed that natural RA and cultured RA can be separated. Results indicated that the mapping and molar ratios of saccharide compositions of the cultured and natural RA samples were different both in cytoplasm and cell wall. The molar ratio of mannose to arabinose of Fraction A was higher than 3.5:1 in cytoplasm in cultured RA, where the ratio was less than 3.5:1 in natural RA. The ratio of the content of arabinose of Fraction D to that of Fraction C was more than 0.5:1 in polysaccharides of cell wall in cultured RA, whereas the ratio was less than 0.5:1 in natural RA. Thus, these ratios may be used to distinguish the growth patterns of RA.

The difference in monosaccharide composition and carbohydrate content between natural RA and cultured RA were compared by GC-MS. The molar ratios of different monosaccharides between natural and cultured RA were also determined. This research not only provides a basis for the identification of RA in terms of growth patterns, but also for evaluation of the quality of Chinese herbal medicines.

## Supplementary Materials

Supplementary materials can be accessed at: http://www.mdpi.com/1420-3049/20/09/16466/s1.

## Abbreviations

RA, Astragalus Radix; MG, *A. membranaceus* var. *mongholicus* (Bge.), and MJ, *A. membranaceus* (Fisch.) Bge., are the two representative species of RA; GC-MS, gas chromatography–mass spectrometry; EPG, endopolygalacturonase; TFA, trifluoroacetic acid.
