# Supplementary material for: Identification of Cultured and Natural Astragalus Root Based on Monosaccharide Mapping
Source: Molecules. 2015 Sep 11;20(9):16466–90. doi: 10.3390/molecules200916466 (PMC6331963; doi:10.3390/molecules200916466)
Supplement: Supplementary file 1 [file molecules-20-16466-s001.pdf]

Supporting Information

Table S1. Monosaccharide contents of Fraction A from different RA samples ($\overline{X} \pm SD$) (mg/g).

Varieties / Content / No.	Fraction A				
	**Glucose**	**Fructose**	**Inositol**	**Sorbitol**	**Galactitol**
**1**	0.553 ± 0.090	0.469 ± 0.002	2.668 ± 0.230	2.346 ± 0.171	2.261 ± 0.170
**2**	0.022 ± 0.017	0.076 ± 0.020	0.338 ± 0.250	0.085 ± 0.031	0.184 ± 0.021
**3**	0.210 ± 0.020	0.026 ± 0.006	3.444 ± 0.390	0.789 ± 0.104	3.305 ± 0.900
**4**	1.595 ± 0.074	0.308 ± 0.090	15.739 ± 5.800	1.856 ± 0.134	8.999 ± 0.180
**5**	0.994 ± 0.080	0.102 ± 0.018	10.096 ± 0.1805	2.414 ± 0.142	3.512 ± 0.800
**6**	0.517 ± 0.033	ND	22.774 ± 0.550	4.256 ± 0.075	14.91 ± 0.012
**7**	7.236 ± 0.350	1.855 ± 0.500	86.481 ± 9.700	8.004 ± 0.102	52.532 ± 0.400
**8**	1.933 ± 0.710	1.323 ± 0.130	33.567 ± 1.300	27.976 ± 0.144	42.429 ± 0.800
**9**	2.177 ± 0.920	1.161 ± 0.060	46.315 ± 1.100	3.933 ± 0.146	34.564 ± 0.140
**10**	1.509 ± 0.194	0.752 ± 0.060	28.492 ± 5.100	7.421 ± 0.116	10.553 ± 0.230
**11**	3.708 ± 0.310	1.131 ± 0.050	74.842 ± 4.800	2.955 ± 0.102	5.346 ± 0.370
**12**	2.792 ± 0.040	ND	37.028 ± 4.200	2.954 ± 0.100	6.599 ± 0.500
**13**	0.051 ± 0.006	0.083 ± 0.000	0.842 ± 0.034	0.260 ± 0.043	1.035 ± 0.120
**14**	0.181 ± 0.023	0.143 ± 0.010	4.117 ± 0.010	0.250 ± 0.102	2.456 ± 0.350
**15**	0.804 ± 0.110	0.454 ± 0.000	1.529 ± 0.140	1.309 ± 0.470	1.800 ± 0.110
**16**	0.248 ± 0.057	0.102 ± 0.012	3.363 ± 0.130	1.433 ± 0.133	4.092 ± 0.160
**17**	0.097 ± 0.112	0.056 ± 0.030	1.160 ± 0.280	0.467 ± 0.101	0.447 ± 0.210
**18**	0.558 ± 0.131	0.248 ± 0.070	10.331 ± 0.790	0.465 ± 0.122	8.541 ± 0.020
**19**	4.705 ± 0.253	0.203 ± 0.085	45.472 ± 0.200	4.930 ± 0.094	67.719 ± 0.600
**20**	4.690 ± 0.340	ND	32.051 ± 0.420	3.937 ± 0.102	28.616 ± 0.110
**21**	3.767 ± 0.220	2.514 ± 0.000	90.073 ± 3.200	9.077 ± 0.104	58.83 ± 0.300
**22**	3.281 ± 0.034	1.668 ± 0.200	46.249 ± 0.300	5.354 ± 0.056	35.124 ± 6.130
**23**	5.039 ± 0.187	1.758 ± 0.470	87.085 ± 2.400	21.162± 095	36.454 ± 3.130
**24**	1.325 ± 0.130	0.698 ± 0.055	35.997 ± 4.300	26.217 ± 0.720	18.562 ± 1.11

ND: Not detected. **1**–**24** stand for different RA samples, as shown in Table 8.

Table S2. Monosaccharide contents of Fraction B from different RA samples ($\overline{X} \pm SD$) (mg/g).

Varieties / Content / No.	Fraction B						
	**Xylose**	**Fucose**	**Rhamnose**	**Arabinose**	**Galactose**	**Mannose**	**Glucose**
**1**	0.169 ± 0.001	0.121 ± 0.001	ND	1.158 ± 0.004	1.713 ± 0.001	11.294 ± 1.010	11.233 ± 0.990
**2**	1.878 ± 0.038	ND	ND	2.34 ± 0.016	3.327 ± 0.314	10.446 ± 1.330	12.247 ± 2.021
**3**	0.244 ± 0.244	0.021 ± 0.021	ND	1.406 ± 0.081	2.301 ± 0.301	11.786 ± 1.786	11.813 ± 0.363
**4**	0.065 ± 0.008	ND	ND	1.082 ± 0.031	0.032 ± 0.008	10.443 ± 0.014	13.195 ± 2.800
**5**	0.169 ± 0.015	0.121 ± 0.002	ND	1.158 ± 0.001	2.713 ± 0.502	11.294 ± 0.071	11.233 ± 1.010
**6**	1.721 ± 0.009	0.395 ± 0.001	ND	1.927 ± 0.038	ND	11.368 ± 0.018	19.592 ± 0.015
**7**	0.549 ± 0.003	ND	0.386 ± 0.001	7.544 ± 0.039	2.604 ± 0.044	24.807 ± 0.735	28.758 ± 0.159
**8**	0.690 ± 0.034	ND	0.597 ± 0.039	11.137 ± 0.069	3.328 ± 0.285	19.358 ± 0.358	31.516 ± 0.055
**9**	1.011 ±0.033	ND	0.928 ± 0.074	5.495 ± 0.052	1.054 ± 0.034	40.576 ± 0.741	43.108 ± 0.115
**10**	ND	0.317 ± 0.013	ND	4.215 ± 0.107	3.285 ± 0.139	19.920 ± 0.099	17.751 ± 0.110
**11**	2.209 ± 0.052	ND	ND	7.686 ± 0.036	2.005 ± 0.038	7.514 ± 0.113	10.164 ± 0.021
**12**	1.476 ± 0.034	ND	1.125 ± 0.078	7.071 ± 0.041	6.777 ± 0.117	10.026 ± 0.458	22.995 ± 0.159
**13**	0.139 ± 0.009	ND	ND	ND	0.298 ± 0.024	10.158 ± 1.900	10.157 ± 0.590
**14**	0.135 ± 0.010	ND	ND	1.213 ± 0.035	0.202 ± 0.010	10.228 ± 1.093	10.375 ± 0.101
**15**	0.303 ± 0.016	ND	ND	1.509 ± 0.001	0.850 ± 0.030	10.202 ± 1.180	10.609 ± 0.140
**16**	0.040 ± 0.012	ND	ND	1.694 ± 0.142	ND	10.683 ± 0.340	10.537 ± 0.332
**17**	0.351 ± 0.040	0.067 ± 0.004	ND	1.499 ± 0.007	0.516 ± 0.044	10.185 ± 1.010	10.767 ± 2.001
**18**	0.074 ± 0.008	ND	0.012 ± 0.005	ND	ND	10.358 ± 0.490	10.262 ± 1.057
**19**	0.176 ± 0.004	ND	0.157 ± 0.005	5.206 ± 0.010	1.227 ± 0.053	13.623 ± 0.133	13.747 ± 0.077
**20**	0.738 ± 0.010	ND	0.679 ± 0.001	10.384 ± 0.000	4.352 ± 0.002	30.232 ± 0.071	29.785 ± 0.001
**21**	0.550 ± 0.023	0.738 ± 0.018	ND	6.775 ± 0.003	2.324 ± 0.121	14.881 ± 0.012	16.723 ± 0.037
**22**	0.127 ±0.002	ND	0.054 ± 0.054	2.932 ± 0.186	0.198 ± 0.198	5.471 ± 5.471	5.736±1.147
**23**	ND	ND	0.165 ± 0.027	2.517 ± 0.004	2.193 ± 0.011	8.863 ± 0.052	9.274 ± 0.024
**24**	0.108 ± 0.005	0.553 ± 0.026	0.191 ± 0.020	1.724 ± 0.024	ND	5.045 ± 0.035	5.273 ± 0.062

ND: Not detected. **1**–**24** stand for different RA samples, as shown in Table 8.

Table S3. Monosaccharide contents of Fraction C from different RA samples ($\overline{X} \pm SD$) (μg/g).

Varieties / Content / No.	Fraction C						
	**Rhamnose**	**Fucose**	**Arabinose**	**Xylose**	**Mannose**	**Glucose**	**Galactose**
**1**	0.017 ± 0.0034	0.0072 ± 0.0001	1.6826 ± 0.2246	2.493 ± 0.177	0.1966 ± 0.0189	0.4674 ± 0.0279	0.5806 ± 0.0262
**2**	0.0122 ± 0.0017	0.0073 ± 0	1.3649 ± 0.1123	2.2427 ± 0.0885	0.2233 ± 0.0094	0.5068 ± 0.0139	0.5435 ± 0.0131
**3**	0.004 ± 0.0015	0.0071 ± 0.0003	0.939 ± 0.3752	1.4152 ± 0.4696	0.0536 ± 0.0411	0.2453 ± 0.0646	0.2404 ± 0.1334
**4**	0.0146 ± 0.0058	0.0072 ± 0.0001	1.5238 ± 0.3012	2.3679 ± 0.5851	0.21 ± 0.12	0.4871 ± 0.1849	0.5621 ± 0.2144
**5**	0.0081 ± 0.0017	0.0068 ± 0.0001	1.1519 ± 0.1123	1.829 ± 0.0885	0.1384 ± 0.0094	0.3761 ± 0.0139	0.3919 ± 0.0131
**6**	0.0128 ± 0.0004	0.0072 ± 0.0001	1.1321 ± 0.1646	2.0876 ± 0.1097	0.1765 ± 0.0331	0.3876 ± 0.0843	0.4872 ± 0.0398
**7**	0.0096 ± 0.0005	0.5691 ± 0.1215	30.026 ± 4.6707	0.153 ± 0.0242	0.0486 ± 0.0087	0.0635 ± 0.004	0.5815 ±0.113
**8**	0.0103 ± 0.0002	0.3972 ± 0.0608	23.4207 ± 2.3353	0.1188 ± 0.0121	0.0363 ± 0.0043	0.0579 ± 0.002	0.4217 ± 0.0565
**9**	0.0099 ± 0.0001	0.4832 ± 0.0304	26.7233 ± 1.1677	0.1359 ± 0.006	0.0425 ± 0.0022	0.0607 ± 0.001	0.5016 ± 0.0283
**10**	0.0098 ± 0.0004	0.5261 ± 0.0911	28.3747 ± 3.503	0.1445 ± 0.0181	0.0455 ± 0.0065	0.0621 ± 0.003	0.5415 ± 0.0848
**11**	0.0101 ± 0.0006	0.4402 ± 0.0833	25.072 ± 3.683	0.1274 ± 0.017	0.0394 ±0.0021	0.0593 ± 0.004	0.4616 ± 0.0236
**12**	0.0087 ± 0.0002	0.4513 ± 0.0608	24.8175 ± 2.3353	0.129 ± 0.0121	0.0456 ± 0.0043	0.0578 ± 0.002	0.5481 ± 0.0565
**13**	0.0244 ± 0.0057	0.0376 ± 0.0111	5.2165 ± 0.9252	1.9366 ± 0.2346	0.1921 ± 0.011	0.7987 ± 0.0126	1.1599 ± 0.1264
**14**	0.0163 ± 0.0028	0.0219 ± 0.0056	3.908 ± 0.4626	1.6048 ± 0.1173	0.1765 ± 0.0055	0.7809 ± 0.0063	0.9812 ± 0.0632
**15**	0.0204 ± 0.0014	0.0298 ± 0.0028	4.5623 ± 0.2313	1.7707 ± 0.0587	0.1843 ± 0.0028	0.7898 ± 0.0031	1.0706 ± 0.0316
**16**	0.0224 ± 0.0043	0.0337 ± 0.0083	4.8894 ± 0.6939	1.8537 ± 0.176	0.1882 ± 0.0083	0.7943 ± 0.0094	1.1152 ± 0.0948
**17**	0.0183 ± 0.0032	0.0258 ± 0.0071	4.2351 ± 0.6299	1.6878 ± 0.0436	0.1804 ± 0.0035	0.7854 ± 0.0017	1.0259 ± 0.0406
**18**	0.0198 ± 0.0028	0.0276 ± 0.0056	4.3257 ± 0.4626	1.875 ± 0.1173	0.1872 ± 0.0055	0.7963 ± 0.0063	1.1025 ± 0.0632
**19**	0.0093 ± 0.001	0.7262 ± 0.0596	35.631 ± 3.7537	0.2195 ± 0.0204	0.0144 ± 0.0071	0.0759 ± 0.0007	0.7327 ± 0.035
**20**	0.0079 ± 0.0004	0.6419 ± 0.0161	30.3224 ± 2.7867	0.1907 ± 0.0135	0.0244 ± 0.0042	0.0769 ± 0.0004	0.6832 ±0.0251
**21**	0.0088 ± 0.0002	0.7035 ± 0.0184	31.69 ± 1.3933	0.2005 ± 0.0067	0.0204 ± 0.0021	0.0765 ± 0.0002	0.6972 ± 0.0125
**22**	0.009± 0.0007	0.7001 ± 0.0482	33.6605 ± 3.2702	0.21 ± 0.0169	0.0174 ± 0.0057	0.0762 ± 0.0006	0.715 ± 0.03
**23**	0.0083 ± 0.0005	0.658 ± 0.0387	31.0062 ± 2.5544	0.1956 ± 0.0055	0.0224 ± 0.003	0.0767 ± 0.0017	0.6902 ± 0.0563
**24**	0.0086 ± 0.0006	0.6714 ± 0.0435	32.0185 ± 0.9671	0.2273 ± 0.0069	0.0186 ± 0.0028	0.0735 ± 0.0003	0.6531 ± 0.0099

1–**24** stand for different RA samples, as shown in Table 8.

Table S4. Monosaccharide contents of Fraction D from different RA samples ($\overline{X} \pm SD$) (μg/g).

Varieties / Content / No.	Fraction D						
	**Rhamnose**	**Fucose**	**Arabinose**	**Xylose**	**Mannose**	**Glucose**	**Galactose**
**1**	0.0398 ± 0.0065	ND	4.1071 ± 0.5505	0.0228 ± 0.0007	0.0566 ± 0.0069	0.4023 ± 0.0223	0.3571 ± 0.0281
**2**	0.0306 ± 0.004	ND	3.3286 ± 0.2755	0.0238 ± 0.0005	0.0468 ± 0.0037	0.4338 ± 0.0111	0.3968 ± 0.014
**3**	0.0352 ± 0.0049	ND	3.7179 ± 0.4129	0.0233 ± 0.0005	0.0517 ± 0.0052	0.4181 ± 0.0167	0.377 ± 0.0211
**4**	0.0342 ± 0.0033	ND	3.7175 ± 0.2752	0.0235 ± 0.0004	0.0513 ± 0.0035	0.418 ± 0.0111	0.377 ± 0.014
**5**	0.0329 ± 0.0025	ND	3.5232 ± 0.275	0.0236 ± 0.0002	0.0493 ± 0.0032	0.4259 ± 0.0112	0.3869 ± 0.014
**6**	0.0317 ± 0.0008	ND	3.8273 ± 0.3526	0.023 ± 0.0006	0.0479 ± 0.0008	0.4132 ± 0.0146	0.3782 ± 0.0132
**7**	0.032 ± 0.0035	0.0032 ± 0.0012	1.0717 ± 0.1776	0.0194 ± 0.0023	0.0235 ± 0.0023	0.3633 ± 0.0116	0.359 ± 0.0122
**8**	0.027 ± 0.0018	0.0049 ± 0.0006	1.3229 ± 0.0888	0.0161 ± 0.0012	0.0202 ± 0.0012	0.3469 ± 0.0058	0.3417 ± 0.0061
**9**	0.0295 ± 0.0009	0.0041 ± 0.0003	1.1973 ± 0.0444	0.0178 ± 0.0006	0.0219 ± 0.0006	0.3551 ± 0.0029	0.3504 ± 0.0031
**10**	0.0308 ± 0.0027	0.0036 ± 0.0009	1.1345 ± 0.1332	0.0186 ± 0.0018	0.0227 ± 0.0018	0.3592 ± 0.0087	0.3547 ±0.0092
**11**	0.0283 ± 0.0022	0.0045 ± 0.0007	1.2601 ± 0.1177	0.0169 ± 0.0027	0.021 ± 0.0006	0.351 ± 0.004	0.346 ± 0.0144
**12**	0.0289 ± 0.0018	0.0042 ± 0.0006	1.2381 ± 0.0888	0.0156 ± 0.0012	0.0243 ±0.0012	0.3577 ± 0.0058	0.3386 ± 0.0061
**13**	0.0167 ± 0.0011	ND	3.4773 ± 0.1853	0.0096 ± 0.0013	0.0277 ± 0.0011	0.3136 ± 0.0603	0.2957 ± 0.0573
**14**	0.0182 ± 0.0005	ND	3.7394 ± 0.0927	0.0115 ± 0.0007	0.0292 ± 0.0005	0.3989 ± 0.0302	0.3767 ± 0.0286
**15**	0.0175 ± 0.0003	ND	3.6084 ± 0.0463	0.0106 ± 0.0003	0.0285 ± 0.0003	0.3563 ± 0.0151	0.3362 ± 0.0143
**16**	0.0171 ± 0.0101	ND	3.5428 ± 1.6455	0.0101 ± 0.0071	0.0281 ± 0.0147	0.3349 ± 0.1768	0.316 ± 0.1745
**17**	0.031 ± 0.0093	ND	5.8043 ± 0.9616	0.0196 ± 0.0043	0.0486 ± 0.0078	0.5637 ± 0.0778	0.5425 ± 0.1357
**18**	0.0298 ± 0.0005	ND	4.8372 ± 0.0927	0.0157 ± 0.0007	0.0387 ± 0.0005	0.4236 ± 0.0302	0.4876 ± 0.0286
**19**	0.0928 ± 0.0109	0.0124 ± 0.0009	15.2504 ± 3.9705	0.0443 ± 0.0003	0.0812 ± 0.0359	1.2461 ± 0.0806	1.2146 ± 0.0828
**20**	0.0774 ± 0.0054	0.0111 ± 0.0005	9.6353 ± 1.9852	0.0439 ± 0.0001	0.1319 ± 0.0179	1.3601 ± 0.0403	1.3317 ± 0.0414
**21**	0.0851 ± 0.0027	0.0118 ± 0.0002	12.4429 ± 0.9926	0.0441 ± 0.0001	0.1066 ±0.009	1.3031 ± 0.0202	1.2732 ± 0.0207
**22**	0.089 ± 0.0082	0.0121 ± 0.0007	13.8466 ± 2.9779	0.0442 ± 0.0002	0.0939 ±0.0269	1.2746 ± 0.0605	1.2439 ± 0.0621
**23**	0.0813 ± 0.0144	0.0114 ± 0.0011	11.0391 ± 3.0245	0.044 ± 0.0037	0.1192 ± 0.0121	1.3316 ± 0.075	1.3024 ± 0.0497
**24**	0.0724 ± 0.0054	0.0108 ± 0.0005	10.9731 ± 1.9852	0.0391 ± 0.0001	0.0983 ± 0.0179	1.3521 ± 0.0403	1.2849 ± 0.0414

ND: Not detected. **1**–**24** stand for different RA samples, as shown in Table 8.
